# Association between germline variants and somatic mutations in colorectal cancer

**DOI:** 10.1038/s41598-022-14408-2

**Published:** 2022-06-17

**Authors:** Richard Barfield, Conghui Qu, Robert S. Steinfelder, Chenjie Zeng, Tabitha A. Harrison, Stefanie Brezina, Daniel D. Buchanan, Peter T. Campbell, Graham Casey, Steven Gallinger, Marios Giannakis, Stephen B. Gruber, Andrea Gsur, Li Hsu, Jeroen R. Huyghe, Victor Moreno, Polly A. Newcomb, Shuji Ogino, Amanda I. Phipps, Martha L. Slattery, Stephen N. Thibodeau, Quang M. Trinh, Amanda E. Toland, Thomas J. Hudson, Wei Sun, Syed H. Zaidi, Ulrike Peters

**Affiliations:** 1 Department of Biostatistics and Bioinformatics, Duke University, 11028A Hock Plaza, 2424 Erwin Road Suite 1106, Durham, NC 27705 USA; 2 Public Health Sciences Division, Fred Hutchinson Cancer Research Center, Seattle, WA USA; 3 National Human Genome Research Institute, National Institutes of Health, Bethesda, MD USA; 4 Institute of Cancer Research, Department of Medicine I, Medical University Vienna, Vienna, Austria; 5 Colorectal Oncogenomics Group, Department of Clinical Pathology, The University of Melbourne, Parkville, VIC 3010 Australia; 6 University of Melbourne Centre for Cancer Research, Victorian Comprehensive Cancer Centre, Parkville, VIC 3010 Australia; 7 Genomic Medicine and Family Cancer Clinic, The Royal Melbourne Hospital, Parkville, VIC Australia; 8 Department of Epidemiology and Population Science, Albert Einstein College of Medicine, Bronx, NY USA; 9 Center for Public Health Genomics, University of Virginia, Charlottesville, VA USA; 10 Lunenfeld Tanenbaum Research Institute, Mount Sinai Hospital, University of Toronto, Toronto, ON Canada; 11 Department of Medical Oncology, Dana-Farber Cancer Institute and Harvard Medical School, Boston, MA USA; 12 The Broad Institute of MIT and Harvard, Cambridge, MA USA; 13 Department of Medical Oncology and Therapeuytic, University of Southern California, Los Angeles, CA USA; 14 Department of Biostatistics, University of Washington, Seattle, WA USA; 15 Oncology Data Analytics Program, Catalan Institute of Oncology-IDIBELL, L’Hospitalet de Llobregat, Barcelona, Spain; 16 CIBER Epidemiología Y Salud Pública (CIBERESP), Madrid, Spain; 17 Department of Clinical Sciences, Faculty of Medicine, University of Barcelona, Barcelona, Spain; 18 ONCOBEL Program, Bellvitge Biomedical Research Institute (IDIBELL), L’Hospitalet de Llobregat, Barcelona, Spain; 19 School of Public Health, University of Washington, Seattle, WA USA; 20 Program in MPE Molecular Pathological Epidemiology, Department of Pathology, Brigham and Women’s Hospital, Harvard Medical School, Boston, MA USA; 21 Cancer Immunology Program, Dana-Farber Harvard Cancer Center, Boston, MA USA; 22 Department of Epidemiology, Harvard T.H. Chan School of Public Health, Boston, MA USA; 23 Department of Epidemiology, Fred Hutchinson Cancer Research Center, University of Washington, 1100 Fairview Ave N, Mail Stop M4-B402, Seattle, WA 98109 USA; 24 Department of Internal Medicine, University of Utah, Salt Lake City, UT USA; 25 Division of Laboratory Genetics, Department of Laboratory Medicine and Pathology, Mayo Clinic, Rochester, MN USA; 26 Ontario Institute for Cancer Research, Toronto, ON Canada; 27 Departments of Cancer Biology and Genetics and Internal Medicine, Comprehensive Cancer Center, The Ohio State University, Columbus, OH USA; 28 Department of Biostatistics, University of North Carolina, Chapel Hill, NC USA

**Keywords:** Colorectal cancer, Cancer genetics, Sequencing

## Abstract

Colorectal cancer (CRC) is a heterogeneous disease with evidence of distinct tumor types that develop through different somatically altered pathways. To better understand the impact of the host genome on somatically mutated genes and pathways, we assessed associations of germline variations with somatic events via two complementary approaches. We first analyzed the association between individual germline genetic variants and the presence of non-silent somatic mutations in genes in 1375 CRC cases with genome-wide SNPs data and a tumor sequencing panel targeting 205 genes. In the second analysis, we tested if germline variants located within previously identified regions of somatic allelic imbalance were associated with overall CRC risk using summary statistics from a recent large scale GWAS (n≃125 k CRC cases and controls). The first analysis revealed that a variant (rs78963230) located within a CNA region associated with TLR3 was also associated with a non-silent mutation within gene *FBXW7*. In the secondary analysis, the variant rs2302274 located in *CDX1*/*PDGFRB* frequently gained/lost in colorectal tumors was associated with overall CRC risk (OR = 0.96, *p* = 7.50e-7). In summary, we demonstrate that an integrative analysis of somatic and germline variation can lead to new insights about CRC.

## Introduction

Colorectal cancer (CRC) is a leading cause of global cancer mortality^1^ and it is estimated in the United States alone that it accounted for nearly 145,600 new cases and 51,020 deaths in 2019^2^. CRC is a biologically heterogeneous disease with multiple tumor subtypes that develop through diverse neoplastic pathways^3^. These characteristics include genetic and epigenetic alterations in multiple driver genes and copy number changes leading to allelic imbalance. The Cancer Genome Atlas (TCGA) Project enabled detailed characterization by identifying a larger number of mutated genes in colorectal tumors, including well known genes, such as *APC, TP53, SMAD4* and *PIK3CA* as well as some that are less well known, such as *SOX9* or *ACVR1B*^4^. A study by our group added additional putative driver genes, such as *PRKCI*, *MAP2K4*, and *TGFBR2 *^5^*.* These results highlighted the importance of several key pathways, including MAPK, WNT and TGFβ-signaling pathways. These detailed molecular data now allow us to better define tumor subtypes, e.g. by somatically mutated pathways and lead to a better understanding of the underlying disease mechanisms.

Meanwhile, substantial progress has been made to identify germline genetic risk factors for overall CRC risk^6–9^. However, there has been less attention given to understanding how germline variants may influence specific somatic mutated genes and pathways. Such studies of germline-somatic relationships could improve our understanding of the underlying etiologic pathways that result in different molecular subtypes of CRC. The work by Carter et al.^10^, is one of the few studies that assessed the associations between somatic mutations and germline variants. Testing relationships between germline variants and somatic mutations in cancer genes across different cancer types within TCGA^11,12^, they highlighted several novel relationships demonstrating the utility of assessing germline and somatic data within the same individuals. Other approaches have involved more of a targeted analysis of known germline variants, using gene expression, examining mutational signatures, or performing pathway analysis^13–19^.

Another approach to elucidate associations between somatic and germline variations is to study if somatically modified regions that are linked to cancer also harbor germline genetic variants associated with CRC. For instance, Palin et al.^12^ examined allelic imbalances in 1,699 CRC cases and highlighted 37 unique regions that were targeted for somatic copy number amplifications (CNA). These regions of allelic imbalances may carry germline risk variants that impact CRC risk, which may be amplified through copy number changes. Performing targeted analyses of germline variants within these CNA regions can decrease the multiple testing burden and highlight variants with an a priori functional interpretation.

**Figure 1:**
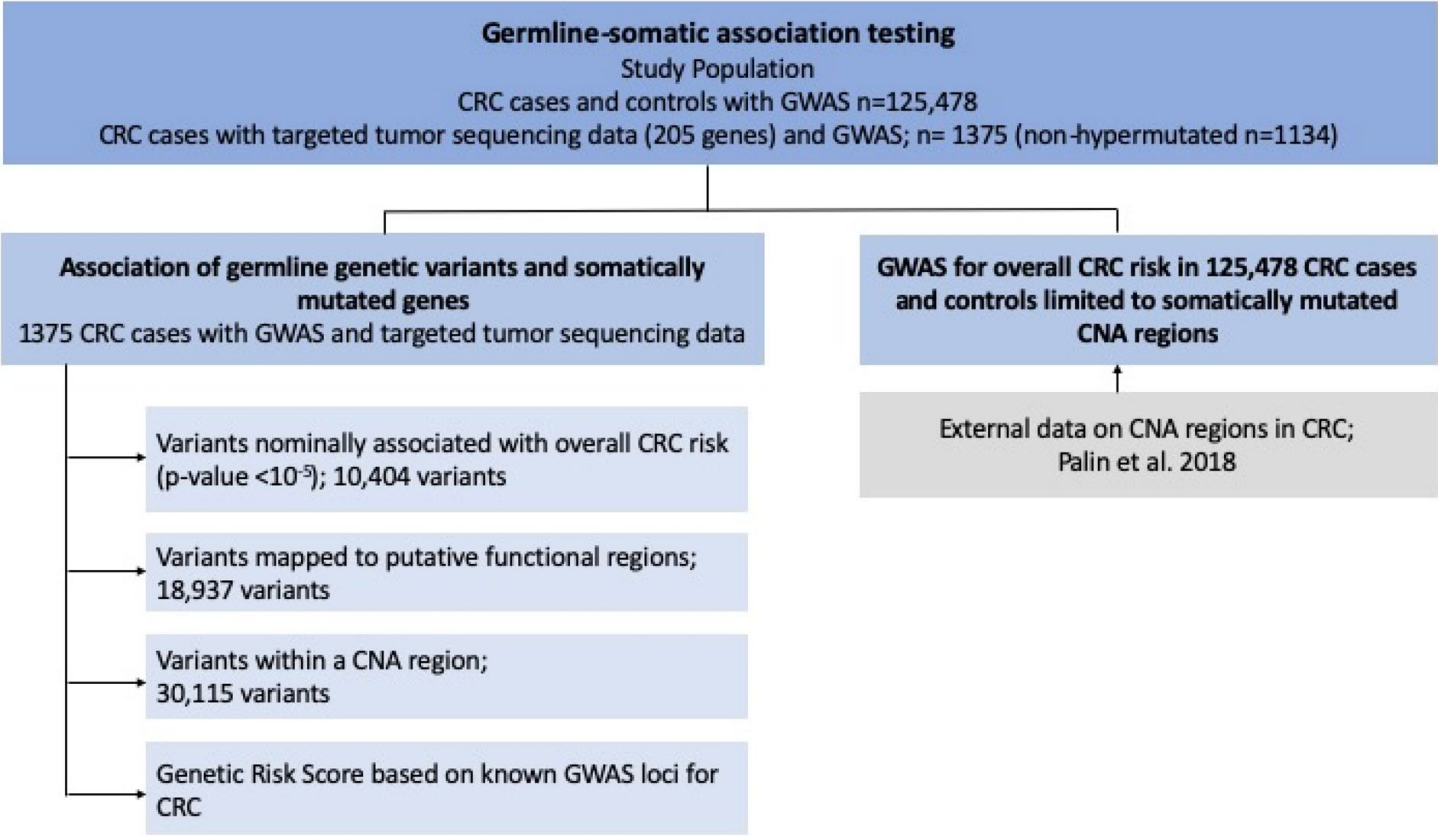
Description of study and pipeline of analysis.

In this paper, we performed a systematic analysis of the relationship between germline variants and somatic events utilizing our large consortium with germline and somatic data. This was done via two separate but synergistic analytical approaches (Fig. 1). In the first analysis, we utilized individual level data from CRC cases with both germline genetic data and somatic mutation data from targeted tumor sequencing^5^ (n = 1375) to test for association between germline genetic variants and having at least one somatic mutation in the gene (SNV or indel). In the second analysis, we utilized our much larger GWAS data (125,478 participants) and conducted a focused association of germline genetic variants with CRC risk in genomic regions that had been identified to carry somatic copy number amplification in CRC^12^.

## Methods

This study was conducted within the Genetics and Epidemiology of Colorectal Cancer Consortium (GECCO) and the Colon Cancer Family Registry (CCFR)^9^. GECCO is an international consortium with data on over 125,000 participants across North America, Asia, Australia, and Europe. The CCFR is a consortium of six centers, consisting of information from approximately 42,500 study participants. For this study, we selected CCFR tumor samples from the Ontario and Seattle CCFR sites^5^. The Institutional Review Board at Fred Hutch, Emory University Institutional Review Board, Mount Sinai Hospital Research Ethics Board, Health Science Research Ethics Board at University of Toronto, Research Ethics Board at the Institute of Cancer Research, Ethics Commission Board at Medical University of Vienna, and Ethics Committee of the Medical Faculty of Heidelberg, approved the study, and all patients provided written informed consent to allow the collection of specimens and data used in this analysis. The study was performed in compliance with the relevant regulations and guidelines.

### Targeted tumor sequencing

Details on the targeted sequencing have been provided in our group’s previous manuscript and in the supplementary methods^5^. Briefly, we developed an AmpliSeq panel of 205 genes that were primarily selected from whole exome sequencing analysis of 1225 CRC cases as well as from literature review^11,20,21^. This panel included some known tumor suppressors and oncogenes (Supplementary Table 1). We obtained tumor DNA using the QIAamp DNA Mini or DNA formalin-fixed paraffin-embedded (FFPE) tissue kits from FFPE sections. We used matched normal DNA isolated from blood, buccal, saliva, or in a small subset (~ 4.5%), adjacent normal colonic FFPE tissues to enable identification of germline from somatic mutations. All sequencing was done using Illumina Genome Analyzer operating procedure via their HiSeq2500 technologies.

Point mutations were called as the intersection of Strelka (v1.0.15) and MuTect (v1.1.7) and annotated via ANNOVAR. We filtered on strand bias, read-depth, alternative read-depth, clustered read position and minor allele frequency in the Exome Aggregation Consortium. We removed cases where there was variation in the mutant allele frequency across read clusters. Indels were called if any two of the following: VarScan2 (v2.4.3), VarDict (Feb 2017), and/or Strelka (v1.0.15) deemed as a mutation^22–24^. The mean sequencing coverage of tumor and normal DNA was 857x and 302x, respectively. Hypermutation status was defined as having 23 or more non-synonymous point mutations. This was based on observing two distinct peaks in the distribution of point mutations and selecting the minimum difference between the two peaks (Zaidi et al.^5^, Supplementary Fig. 9). More details are provided in the Supplementary Methods, summarizing the supplementary methods of the group’s previous work.

### Germline variant data

GWAS genotyping data are available in a total of 125,478 CRC cases and controls of which 1375 CRC cases also had targeted tumor sequencing data. The participants and GWAS data have been described in detail elsewhere^25^. Briefly, we excluded individuals with discrepancies between genotyped and reported sex. We calculated identity by descent via KING-robust and removed any duplicate individuals or second degree or more relatives. Additional QC has been described previously^9^. Principal component analysis was done to account for potential population substructure. The top principal components (PCs) were included in downstream analyses. The GWAS data was imputed to Haplotype Reference Consortium (HRC) panel^26^. We restricted our analysis to variants with an imputed allele frequency greater than 1%.

### Statistical analysis

In the first analysis, we examined individual level genome-wide germline genetic data and somatic data from 1375 CRC cases. The outcome was the somatic mutation status whether the CRC case had one or more non-silent point mutation or indel in the gene. We restricted our analyses to genes with a non-silent mutation frequency above 5% as we had limited power to analyze genes less frequently mutated. The germline variants of interest are detailed below and in Fig. 1. We fit logistic regression models to assess for association between germline genetic variants and somatically mutated genes adjusting for age at diagnosis, sex, studies, and the top ten PCs. As the hypermutation status has a major impact on the frequency of mutated genes, we performed two analyses, one in the non-hypermutated samples, and one in the combined sample of both hypermutated and non-hypermutated participants (due to small sample size of hypermutated alone). In the latter analysis, we adjusted for hypermutation status. All analyses were done using the EPACTS software (https://genome.sph.umich.edu/wiki/EPACTS). As conducting a complete GWAS for each mutated gene in only 1375 samples has limited power, we examined the following sets of germline variants for an association with each somatic mutated gene (Fig. 1).

- Variants associated with overall CRC risk with p-value < 1e-5 based on our combined GWAS of 125,478 samples^9^;
- Variants mapped to putative functional regions if variants satisfy any of the following criteria: coding variant in one of the exons of the gene^27,28^, within 1000 bp upstream of the transcription start site (promoter region), within 200 KB of the gene (in any direction) and within a variant enhancer loci (VEL) as defined by Akhtar-Zaidi et al^29^, within 200 KB of the gene and within a known distal promoter/enhancer of that gene in digestive or immunology tissue^30^, over 200 KB of the gene and within the overlap of a distal promoter/ enhancer that was linked to the gene based on gene expression AND in a VEL^29^. For this analysis, we dropped genes AMER1, BCOR, MXRA5 and USP9X as we do not have available GWAS data or functional information on the sex chromosomes;
- Variants located in the 37 somatic CNA regions defined by Palin et al.^12^. If a variant was within one of these regions we examined its association with a somatically mutated gene on the same chromosome.

To account for multiple testing, as well as the strong correlation between variants, we calculated the effective number of independent tests (M_eff_) in each of these sets. This was computationally feasible as the number of variants in each set is well below 100,000 and we further reduce the computational burden by calculating. M_eff_ chromosome by chromosome via Li et al.^31,32^. The LD information was derived from a subset of 8,573 individuals from our data set. The significant threshold for each analysis was set to 0.05/M_eff_.

In addition,we performed a Genetic Risk Score (GRS) analysis of known CRC variants with total tumor mutational burden or having a mutation in a specific pathway (the WNT-signaling, TFGβ, IGF2-PI3K, or DNA Repair and Replication/MMA, Supplement Table 2). Weights were based on the effect size estimated from a recent analysis, with adjustment for winner's curse (Supplement Table 3). Analysis was done via MiST^31,32^ to test for both the effect of the GRS (weighted sum) and for effects not through the GRS.

### Association with overall CRC risk in 125,478 participants limited to somatically mutated CNA regions

We next used our GWAS for overall CRC risk in 125,478 participants focusing on germline genetic variants located in the 37 somatic CNA regions defined by Palin et al^12^ to assess whether or not subsetting variants to those within a CNA region would further reveal additional loci that are associated with CRC. We then evaluated these SNPs associations with overall CRC risk at the p-value cutoff of 0.05/M_eff_, where M_eff_ was calculated based on the number of variants within these CNA regions.

## Results

Among the 1375 CRC cases with available targeted tumor sequencing and germline genetic variant data: 1134 were non-hypermutated and 241 were hypermutated (Table 1). The mean age of diagnosis was 61.6 years and the number of men and women were roughly the same (49.2% men). In the non-hypermutated sample, 12 genes were somatically mutated in at least 5% of cases while in the combined sample (non-hypermutated and hypermutated cases) 62 genes were somatically mutated in at least 5% of the cases (Supplement Table 4). The median number of non-silent mutations per case was 5, 44, and 6 in the non-hypermutated, hypermutated, and combined, respectively (Table 1). We compared our mutational frequency to those in TCGA (Supplementary Methods) and in general saw slightly higher all mutations in our dataset and slightly higher non-silent mutations in the TCGA dataset (Supplementary Table 6, Supplementary Fig. 1).

**Table 1:** Descriptive statistics of the study population with data on germline variants based on genome-wide association study and somatic mutations based on targeted tumor sequencing (n = 1375 colorectal cancer cases).

	Hypermutated (N = 241)	Not Hypermutated (N = 1134)	Overall (N = 1375)
**Sex**			
Female	141 (59%)	558 (49%)	699 (51%)
Male	100 (41%)	576 (51%)	676 (49%)
**Age at diagnosis**			
Mean (SD)	64.0 (12.5)	61.0 (12.0)	61.6 (12.2)
Median [Min, Max]	66.0 [28.0, 90.0]	63.0 [21.0, 91.0]	63.0 [21.0, 91.0]
**Study**			
CORSA	22 (9%)	84 (7%)	106 (8%)
CPSII	54 (22%)	164 (14%)	218 (16%)
OFCCR	91 (38%)	546 (48%)	637 (46%)
SFCCR	74 (31%)	340 (30%)	414 (30%)
**Number non silent indel mutations**			
Mean (SD)	14.7 (11.3)	1.01 (1.40)	3.40 (7.14)
Median [Min, Max]	13.0 [0, 49.0]	1.00 [0, 14.0]	1.00 [0, 49.0]
**Number non silent SNV mutations**			
Mean (SD)	36.5 (39.0)	4.72 (2.45)	10.3 (20.4)
Median [Min, Max]	26.0 [2.00, 334]	5.00 [0, 14.0]	5.00 [0, 334]
**Number non silent indel and SNV Mutations**			
Mean (SD)	51.2 (39.0)	5.73 (3.03)	13.7 (23.9)
Median [Min, Max]	44.0 [2.00, 335]	5.00 [0, 24.0]	6.00 [0, 335]

### Germline-somatic association testing for germline variants nominally associated with overall CRC risk

Based on our previously conducted GWAS^9^ in 125,478 participants,10,404 SNPs (M_eff_:1835) had a p-value < 1e-5. Significance was assessed at the 0.05/(12*1835) = 2.27e-6 in the non-hyper mutated and 0.05/(62*1835) = 4.39e-7 in the combined analyses. No SNP was found to be significantly associated with the presence of non-silent mutations in any gene. The most associated in the combined sample (though not statistically significant) was rs6933790 (6:41672769_T/C) with CUX1 (p-value 4.72e-05, located on chromosome 7) while in the non hypermutated sample it was rs4960622 (7:154631285_C/G) with RYR1 (p-value 3.05e-05, located on chromosome 19).

### Germline-somatic association testing for germline variants mapped to putative functional regions

We restricted the analysis to germline SNPs that were in putative functional regions in or near the somatically mutated gene. We tested on average 327 SNPs per gene. In the combined analysis we examined 18,908 SNPs (M_eff_ = 7613) and in the non-hypermutated we tested 3113 SNPs (M_eff_ = 1304). After adjusting for multiple testing, no germline SNPs within these regions were associated with a somatically mutated gene.

### Germline-somatic association testing for germline variants within a CNA region

We tested for associations of SNPs in CNA regions with somatically mutated genes located on the same chromosome. As these CNA regions are only on a subset of chromosomes, we only assessed 9 of the 12 somatically mutated genes in the non-hypermutated and 44 of the 62 in the combined analysis. In the non-hypermutated sample we assessed 17,721 associations (M_eff_ 4,710, 14,816 unique genomic variants). Variant 4:186990948_A/G, (rs78963230) was significantly associated with the presence of non-silent somatic mutations in gene *FBXW7* (p-value 4.4e-6, odds ratio of 2.19 (95% CI 1.57–3.06), effect allele frequency 0.13). The germline variant is located within the region of allelic imbalance associated with gene *TLR3*^12^. This association remained (though not significant after multiple testing) in the combined analysis of hypermutated and non-hypermutated (odds ratio of 1.79, 95% CI 1.34–2.39) although the signal was weaker (p-value of 9.12e-5). There were 198 people (14.4%) with one or more non-silent mutations in *FBXW7*. This result was not replicated in a sample of 306 non-hypermutated TCGA participants with germline and somatic data (Supplementary Methods, p-value: 0.70, estimated odds ratio of 0.87).

### GRS analysis of tumor burden and known pathways

After adjusting for multiple testing, we found no association (Supplement Table 5). There was a marginally significant association between total tumor analysis and the known loci, likely being driven by the fixed effect of the known variants (i.e. the weights) but this did not remain significant when accounting for multiple testing.

### GWAS for overall CRC risk in 125,478 participants in somatically mutated CNA regions

**Figure 2:**
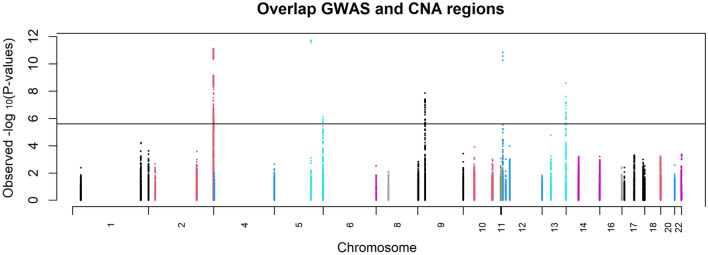
Modified Manhattan plot of areas that overlap with CNA regions and their respective association with CRC risk.

When we restricted the entire GWAS for 125,478 participants to somatic CNA regions for CRC highlighted by Palin et al.^12^ we observed several loci associated with CRC risk. In total, there were 48,037 SNPs (M_eff_: 19,659) that mapped to these 37 regions across seventeen chromosomes. There were 370 variants significant at the 0.05/19,659 = 2.54e-6 threshold. We kept the lead SNP within each window of 1 MB, resulting in 6 loci (Fig. 2, Table 2). Of these 6 loci 5 were previously reported (loci 2q33.1, 5q22.2, 9p21.3, 12p13.32 and 13q22.1^9^) and one novel locus on chromosome 5 (rs2302274, p-value 7.5e-7) and is located at c-14 in the 5 prime UTR of CDX1 mrRNA. This variant was in a CNA region for *CDX1/PDGFRB*^12^. Three of the six variants were located within a VEL (Table 2). In addition, two (rs1537372 and rs45597035) are within 200 KB of the gene promoter (*CDKN2A* and *KLF5*) respectively. The variant rs2302274 was in addition found to be within a distal promoter for gene *CDX1* in digestive or immunology tissue^30^*.* We tested if significant loci occurred more frequently in copy number gain or loss regions, but we did not find a difference (Fisher-Exact test p-value = 0.16, Supplementary Table 7).

**Table 2:** Association between germline genetic variants within somatic copy number amplification regions and colorectal cancer (CRC) risk based on data from over 125,000 CRC cases and controls.

RS number (Chromosome position)	EAF	OR (95% CI)	p-value	Associated gene (Gain/Loss^a^)	Known GWAS locus
rs983402 (2:199781586_T/C)	0.31	1.07 (1.05–1.09)	7.71e-12	*SATB2 (Gain)*	Yes (Huyghe 2019)
rs536830449 (5:112062904_A/G)	0.994	0.54 (0.46–0.64)	1.91e-12	*APC (Loss)*	Yes*(Peters 2010)
**rs2302274**^**b**^** (5:149546426_A/G)**	**0.52**	**0.96 (0.94–0.97)**	**7.50e-7**	***CDX1/PDGFRB (Gain)***	**No**
rs1537372^b^ (9:22103183_G/T)	0.44	0.95 (0.93–0.97)	1.41e-8	*CDKN2B (Loss)*	Yes (Huyghe 2019)
rs35808169 (12:4368607_T/C)	0.81	0.92 (0.90–0.95)	1.46e-11	*CCND2 (Gain)*	Yes (Jia 2013)
rs45597035^b^ (13:73649152_A/G)	0.66	1.06 (1.04–1.08)	2.66e-9	*KLF5 (Gain)*	Yes (Huyghe;Law 2019)

^a^Gain/Loss based on Table 1 of Palin et al. ^b^Within a VEL region

*Within 1 MB of rs755229494 (Niell 2003, Peters 2010, Boursi 2013).

## Discussion

We found one variant located within the CNA region of *TLR3* that was associated with somatic mutations in *FBXW7*. When we overlaid somatic CNA with GWAS results from all 125,478 participants, we found five known GWAS regions and highlighted one novel region located on Chromosome 5 that remained significant after adjusting for multiple comparisons.

**Figure 3:**
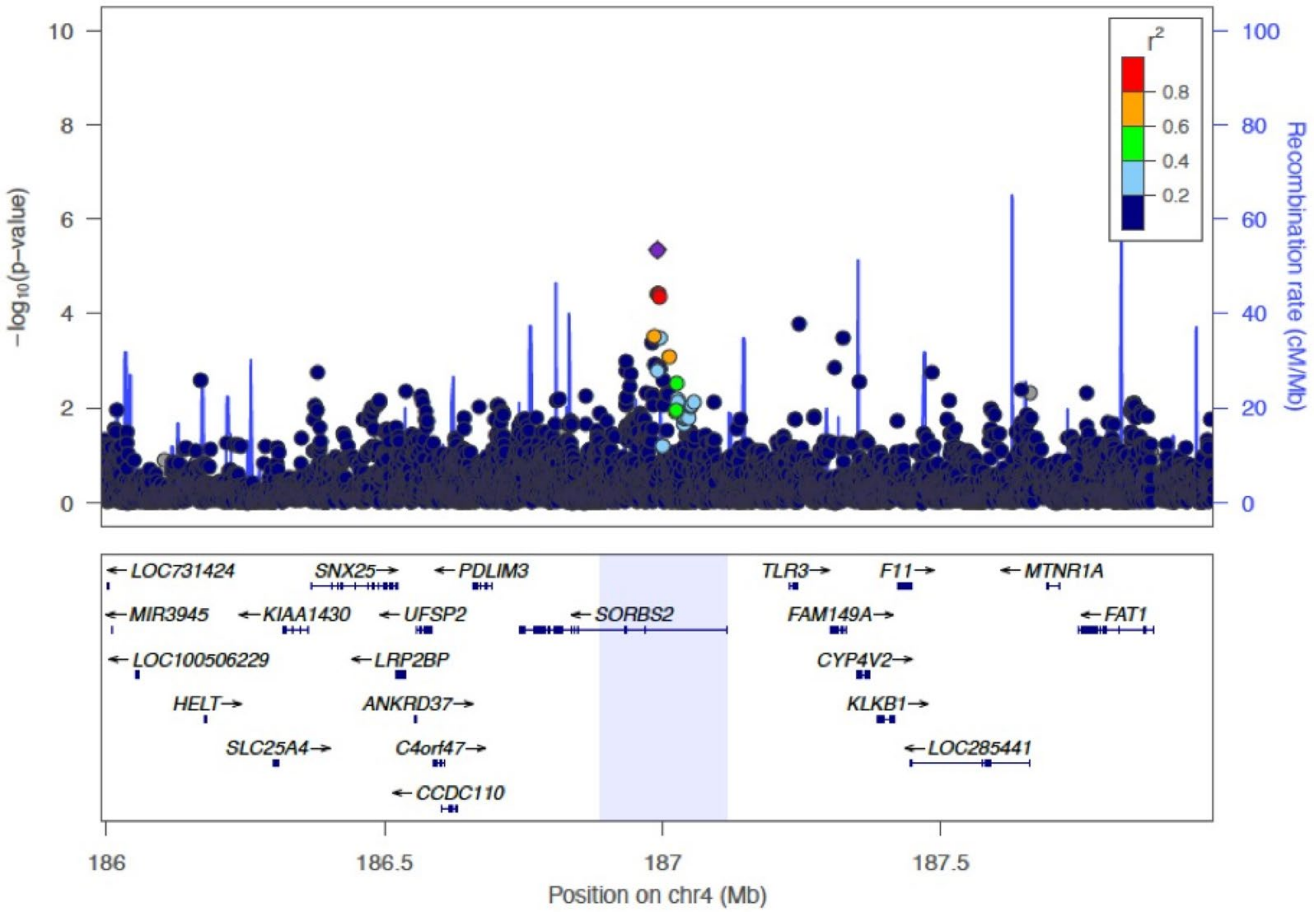
LocusZoom plot of SORBS2 region. Highlighted region shows the CNA region called by Palin et al.

Analyzing SNPs located within somatic CNA regions for CRC, we observed that the variant rs78963230 was associated with a non-silent somatic mutation in *FBXW7* among non-hypermutated cases. This germline variant was located in the region relating to allelic imbalances in gene *TLR3,* and appears to be located within *SORBS2* (Fig. 3 made with LocusZoom^33^). This location was associated with a loss by Palin et al. (Table 1)^12^. This variant is common in European ancestry populations with a MAF of 0.16 (https://gnomad.broadinstitute.org/). rs78963230 is located 34 MB away from the gene body of *FBXW7*. This type of trans-like associations were also observed in Carter et al., though at a larger scale. *SORBS2* has been associated with metastasis in ovarian cancer^34^, survival in a small sample of renal cancer patients^35^, and described as a putative tumor suppressor gene for cervical cancers^36^. Functionally, *FBXW7* is a known tumor suppressor^37,38^ and known to interact with *KLF5*^38^. Protein levels of *FBXW7* have also frequently been found to be lower in CRC tumor tissue in comparison to normal tissue^39–42^. Expression of the gene has been associated with inhibiting the CRC cell migration^39,42–44^. The *TLR3* gene has been reported to be related to worsening pancreatic cancer survival in a small study^45^, and as a potential target for KRAS CRC cases^46^. In lab conditions, FBXW7ɑ appeared to suppress the expression of *TLR4*, suggesting a possible interplay with genes from the same family^47^. Macrophage miR-223 has also been found to modify the relationship between FBXW7 and TLR4^48^. In summary, the observed links between FBXW7 and genes of the toll-like receptor family may help explain the observed association between germline variants in *TLR3* and somatic mutations in *FBXW7.* However, this result did fail to reproduce in a smaller TCGA sample group. This lack of replication could be due to differences in tumor site/collection, age, sequencing depth, or any variety of factors. Larger sample sizes will be needed to assess this relationship.

When we tested germline genetic variants located in regions of somatic allelic imbalance in our GWAS of over 125 thousand participants, we found six loci that were significantly associated with CRC risk. Five of these six were in known loci and located within the following regions of allelic imbalance: *SATB2, APC, CDKN2B, CCND2,* and *KLF5* (Table 2). The one novel locus was located within a gained copy number region for *CDX1/PDGFRB* on chromosome 5^12^. Caudal-type homeobox 1 (*CDX1*) is an intestine-specific transcription factor^49^. CDX1 has been shown to reduce proliferation by blocking β-catenin/T-cell factor transcriptional activity^50^. CDX1 encodes a key regulator of differentiation of enterocytes and its expression is decreased or lost in CRC cell lines and CRC tumor tissue^51,52^. CDX1 (together with CDX2) can function as a tumor suppressor and concomitant loss of CDX1 can significantly increase the incidence of tumors APC(Min/ +)-Cdx2 mice^53^. *PDGFRB* has also been found to be associated with recurrence of CRC^54^ and gastric cancer^55,56^ Overall, these data provide strong support, particularly for *CDX1* as a putative functional candidate gene involved in CRC tumorigenesis.

There are several strengths and limitations of this project. In comparison to existing studies, we have a relatively large sample with available germline and somatic data; however, given the very large number of germline genetic variants across the genome the power remains limited for any agnostic genome-wide association study. To increase statistical power, we thus utilize our very large GWAS of CRC and functionally informed annotations. The selection of putative somatic driver genes that we sequenced in tumors was informed by whole exome sequencing of over 1200 samples, so it is likely that we have captured all common driver genes for CRC. However, we still only have a limited targeted gene panel and were not able to evaluate all somatic mutated genes across the genome; although those would have been infrequently mutated and would have further increased the multiple comparison burden. Another potential limitation is our sole focus on somatic mutations indicating potential altering gene activity in the tumor. For example, Carter et al. found that expression in thyroid tumors was associated with germline variants^10^, in addition methylation is a known potential predictor of CRC status with developed at home tests^57^, and overall loss of methylation has been associated with tumor invasion signatures^58^. In addition, we were not able to assess copy number alterations within our sequencing panel due to technical limitations.

In conclusion, we performed a novel analysis combining germline genetic and somatic data to better understand CRC. Given limited statistical power, we selected SNPs a priori with potential functional annotation and assessed their association with somatic mutations or selected SNPs within regions of tumor CNA imbalance and evaluated their association with CRC risk. As the amount of available data from disparate sources grows, integrative analyses for testing associations will need to be utilized. Future studies will look at potentially replicating the results found here.

## Supplementary Information

Supplementary Information 1.

Supplementary Information 2.

## Data Availability

All data generated or analyzed during this study are included in this published article (and its supplementary information files). The original tumor sequencing data are available at the database of Genotypes and Phenotypes (dbGaP, accession phs002050.v1.p1). The original genotype data have been deposited at dbGaP under accession numbers phs001415.v1.p1, phs001315.v1.p1, phs001078.v1.p1, phs001499.v1.p1, phs001903.v1.p1, and phs001856.v1.p1.

## Abbreviations

CI: Confidence intervals
CNA: Copy number amplification
CRC: Colorectal cancer
EAF: Effective allele frequency
GRS: Genetic risk score
OR: Odds ratio
VEL: Variant enhancer loci
